# Driving Tau Pathogenicity Seeding - How Fibril Structure and Post-Translational Modifications Alter Seeding Capacity

**DOI:** 10.1002/alz.094584

**Published:** 2025-01-09

**Authors:** Alysa Kasen, Libby Breton, Lindsay Meyerdirk, Sofia Lövestam, Jacob McPhail, Ariel Louwrier, Sjors Scheres, Michael Henderson

**Affiliations:** 1 Van Andel Research Institute, GR, MI, USA; 2 MRC Laboratory of Molecular Biology, Cambridge, United Kingdom; 3 StressMarq Biosciences Inc, Victoria, BC, Canada

## Abstract

**Background:**

The accumulation of hyperphosphorylated, aggregated tau in neurons is one of the hallmarks of Alzheimer’s disease (AD). Recent work in structural biology has solved the structure of tau fibrils in several tauopathies and found that the structure of the tau fibrils varies between diseases, but fibril structure is conserved among patients within the same disease, suggesting fibril structure relates to its pathogenicity. Tau fibrils derived from AD brain (AD PHFs) seed AD-like pathology in wild-type mice, yet efforts to recapitulate this seeding with recombinant fibrils have failed. The differential capacity of tau fibrils to seed pathology also supports the relevance of tau structure to its pathogenicity. We hypothesized that recombinant fibrils that recapitulate the core region structure of AD tau will show similar seeding capacity to AD tau, with PTMs playing a modulatory role.

**Methods:**

We took advantage of recently developed recombinant tau fibrils with diverse structures to investigate how tau fibril structure and PTMS are related to tau seeding capacity in primary cortical neurons. Fibrils of interest from the screen were then assessed through hippocampal injection into wild-type and *MAPT* knock-in mice.

**Results:**

In-*vitro* screening showed that fibrils more closely resembling the core structure of an AD PHF had a higher seeding capacity than other fibrils. However, the replication of the core structure alone in truncated fibrils is not sufficient to fully replicate the seeding capacity of AD PHFs, indicating that the region outside of the core structure, containing PTMs, likely plays an essential role in pathology seeding. This finding was replicated in the seeding model into wild-type and *MAPT* KI mice. We also found that tau fibrils containing PTMs had a higher seeding capacity than full length fibrils without PTMs. However, the presence of PTMs alone was not enough to fully replicate the seeding capacity of AD PHFs.

**Conclusions:**

The structure and PTM patterns of tau fibrils appear to be closely tied to fibril pathogenicity, with recombinant fibrils more closely resembling AD PHFs having a more similar seeding capacity. We believe that the generation of fibrils closely resembling AD PHFs can lead to improved model systems of tau pathology in AD.

